# The genome of the migratory nematode, *Radopholus similis*, reveals signatures of close association to the sedentary cyst nematodes

**DOI:** 10.1371/journal.pone.0224391

**Published:** 2019-10-25

**Authors:** Reny Mathew, Charles H. Opperman

**Affiliations:** Department of Entomology and Plant Pathology, North Carolina State University, Raleigh, NC United States of America; INRA, FRANCE

## Abstract

*Radopholus similis*, commonly known as the burrowing nematode, is an important pest of myriad crops and ornamentals including banana (*Musa spp*.*)* and *Citrus spp*. In order to characterize the potential role of putative effectors encoded by *R*. *similis* genes we compared predicted proteins from a draft *R*. *similis* genome with other plant-parasitic nematodes in order to define the suite of excreted/secreted proteins that enable it to function as a parasite and to ascertain the phylogenetic position of *R*. *similis* in the Tylenchida order. Identification and analysis of candidate genes encoding for key plant cell-wall degrading enzymes including GH5 cellulases, PL3 pectate lyases and GH28 polygalactouranase revealed a pattern of occurrence similar to other PPNs, although with closest phylogenetic associations to the sedentary cyst nematodes. We also observed the absence of a suite of effectors essential for feeding site formation in the cyst nematodes. Clustering of various orthologous genes shared by *R*. *similis* with other nematodes showed higher overlap with the cyst nematodes than with the root-knot or other migratory endoparasitic nematodes. The data presented here support the hypothesis that *R*. *similis* is evolutionarily closer to the cyst nematodes, however, differences in the effector repertoire delineate ancient divergence of parasitism, probably as a consequence of niche specialization. These similarities and differences further underscore distinct evolutionary relationships during the evolution of parasitism in this group of nematodes.

## Introduction

Global crop loss caused by plant-parasitic nematodes approaches ~$100 billion annually [1]. Based upon their unique life cycles there exists two main categories of plant parasitic nematodes: ectoparasites and endoparasites. The endoparasites are further classified into two broad groups based on their feeding mechanisms, specifically, sedentary endoparasites and migratory endoparasites. The two most economically important sedentary endoparasitic nematodes are the root knot nematodes *Meloidogyne spp*., and the cyst nematodes belonging to the *Heterodera* and *Globodera spp*. Of these, genome and transcriptome sequences of several cyst nematodes, such as the soybean cyst nematode *H*. *glycines* [2,3] and the potato cyst nematodes, *G*. *pallida*, *G*. *ellingtonae and G*. *rostochiensis*. [4–6] exist in the public domain. Additionally, genomes/transcriptomes of numerous root-knot nematodes, including *M*. *hapla* [7], *M*. *incognita*, *M*. *javanica*, *M*. *arenaria* [8,9], *M*. *graminicola* [10] *M*. *floridensis* [11] and *M*. *enterolobii* [12] have been exploited to reveal important determinants of plant parasitism by sedentary endoparasitic nematodes. These nematodes form specialized feeding sites in plant roots via different mechanisms, known as giant-cells in root-knot species and syncytia in cyst nematode species [13]. Also, more recently, the draft genome of the sedentary semi-endoparasitic nematode species, *R*. *reniformis* has also been released in the public domain [14].

Unlike the sedentary endoparasitic RKN and cyst nematodes and the semi-endoparasitic, *R*. *reniformis* the burrowing nematode *Radopholus similis* is a migratory endoparasite. *Radopholus* is a member of the Pratylenchidae family, which have not been shown to form specialized feeding sites. Although the currently known host range of *R*. *similis* extends to more than 365 plant species [15], *R*. *similis* is an economically significant parasite of *Musa* spp. worldwide [16], and also causes significant damage to *Citrus* spp., coconut, palm, coffee, sugarcane, avocado, black pepper, foliage ornamentals of the family (*Philodendron*, *Anthurium*, *Hibiscus)* and ginger (*Zingiberacieae*). Additionally, bananas and plantains (*Musa sp*.*)* are consumed by ~400 million people worldwide and the export industry for these crops serves as a crucial mode of income for people living in many countries, including Colombia, Ecuador, Costa Rica and the Philippines [17].

*R*. *similis* was first identified in 1893, in the banana growing areas in Fiji islands [18] and since then has reached multiple continents, most likely on infested plant material. Characterization of *R*. *similis* populations from multiple regions worldwide indicated that *R*. *similis* radiation is a relatively recent event, occurring within the past 200–300 years [19–21]. *R*. *similis* thrives in the tropical and the sub-tropical regions around the world including east Africa, south and central America, parts of Asia, Australia and multiple regions in Europe. In the United States, *R*. *similis* is found in Florida, Texas and the islands of Hawaii & Puerto Rico [22]. *R*. *similis* is a sexually dimorphic PPN. Males and females can be classified by the unique sexual dimorphism that exists in their cephalic region, and this trait also supports the hypothesis that *R*. *similis* is closely related to the cyst nematodes, belonging to the ‘Hoplolaimidae’ family [23,24]. *R*. *similis* males are generally 500–600 μm long and possess a raised lip, a feature not shared by the females [25]. Males are more slender than females and possess a degenerate stylet, accounting for their non-feeding nature. Additionally, males possess a characteristic curved spicule near the tail end and a prominent bursa. On the contrary, females possess a distinctive stylet approximately 16–21 μm long with three knobs. *R*. *similis* females are generally 550–800 μm long ending with a unique hyalinated tail region. *R*. *similis* reproduces as a facultative hermaphrodite, although out crossing occurs preferentially when males are present [26].

The life cycle of *R*. *similis* is completed in 18–20 days at a temperature range of 25–30°C, however populations in Europe seem to undergo reproduction even at lower temperatures [23]. Eggs are laid as the females migrate through the root system and females can lay up to 4–5 eggs a day for several weeks. *R*. *similis* juveniles and females are parasitic, with the juveniles feeding outside the root system occasionally. The intercellular migration and feeding by these vermiform nematodes cause significant cell damage, which leads to the characteristic reddish-brown lesions associated with these nematodes. Additionally, damage caused by the tunneling and feeding activities, leaves the root system wounded and susceptible to opportunistic bacteria or fungi that may enter the plant through the wounded site.

The major underground symptoms of an *R*. *similis* infection seen in a banana plant is the characteristic necrotic lesions on the roots, that results in weakened root systems and reduced plant growth due to poor uptake of nutrients and water. This ultimately leads to toppling of banana plants especially during heavy rains and storm conditions and is a major concern among growers in the banana growing areas. Currently a variety of practices such as hot water and/or nematicidal treatment of the corms and suckers of banana plants, guying and propping of fallen banana plants [27] to hold them in place and even resistant cultivars have been developed to combat this parasite. Resistant cultivars of *Musa spp*. such as the Yangambi Km5 and Pisang Jari Buaya(AAA) have been very successful in reducing nematode populations [28] in major banana growing regions in Africa. The resistance in these cultivars has been shown to be mediated by the deposition of phenalenone-type phytoalexins at nematode infected sites [17]. However, these cultivars do not seem to be successful in managing *R*. *similis* populations infecting *Musa spp*. in Uganda, which has been attributed to pathogenicity variability between *R*. *similis* populations [28]. Furthermore, multiple biological agents utilized to control *R*. *similis* on bananas such as the “mutualistic endophyte *Fusarium oxysporum* strain 162” and the “antagonistic bacteria *Bacillus firmus”* have shown efficacy under controlled conditions; however, the protection they offer is generally limited to a single growing season[17,29]. Management strategies utilizing nematicides are restricted, both by cost and by regulations due to toxicity concerns for non-target organisms (including humans) as well as environmental and ground water impacts.

The genome sequence of *R*. *similis* may provide insights into the variety of mechanisms adopted by this pathogen to maintain a plant-parasitic lifestyle. In order to characterize the potential role of putative PPN effectors, including plant cell-wall degrading enzymes, encoded by *R*. *similis* genes we compared predicted proteins from our draft *R*. *similis* genome [30] with other plant-parasitic nematodes in order to define the suite of excreted/secreted proteins that enable it to function as a parasite and occupy new niches; and secondly, to ascertain the phylogenetic position of *R*. *similis* in the Tylenchida order. Small Subunit ribosomal DNA (SSU rDNA) studies have placed *R*. *similis* as a sister taxon of the endoparasitic cyst nematodes [24,31,32]. Using our predicted protein set, we performed phylogenetic analyses of orthologous clusters shared with the cyst nematodes *G*. *rostochiensis*, *G*. *pallida*, *H*. *glycines*, the root-knot nematodes *M*. *hapla*, *M*. *incognita*, the migratory endoparasite *D*. *destructor* and the free-living nematode *C*. *elegans*. We were able to corroborate the SSU rDNA phylogenetic studies that *R*. *similis* is indeed a sister-taxon to the cyst nematodes. In this article, we present key parasitism/effector features of *R*. *similis* and its phylogenetic position in comparison with several nematodes exhibiting different lifestyles.

## Results and discussion

### Parasitism gene repertoire and effectors

PPN effectors are secreted when the PPNs penetrate the host tissues with their spear-like stylet. Most of these effectors are produced primarily in the two subventral and one dorsal gland at different stages of parasitism and play an important role during a nematode-plant interaction. Over the years, many *R*. *similis* effectors and parasitism genes have been reported including β-1,4 endoglucanase [33], endoxylanases [34], transthyretin-like proteins [35] serine carboxypeptidase [36–38] cathepsin-B [38,39] and calreticulin [37]. We mined our predicted protein sequences in search for these known parasitism genes and found that the *R*. *similis* predicted protein set possesses all the known parasitism PPN proteins (Table B in S1 File). Additionally, *R*. *similis* protein sequences were scanned for putative signal peptides using SignalP. Based on this search, 1548 protein sequences were identified which had putative signal peptides. However, only 1405 protein sequences out of the 1548 SignalP identified candidates did not possess a transmembrane domain.

To obtain the effector repertoire encoded by *R*. *similis*, we extracted sequences coding for ~100 effectors from a diverse array of nematodes including the root-knot nematodes such as *M*. *hapla*, *M*. *incognita* and cyst nematodes like *G*. *rostochiensis*, *H*. *avenae*, *G*. *pallida etc*. (Table B in S1 File). We noted that *R*. *similis* does not possess any homologues of the CLAVATA3/ENDOSPERM SURROUNDING REGION(ESR), (CLE)—like peptides present in the cyst nematodes such as *G*. *tabacum*, *G*. *rostochiensis* and *G*. *virginiae*, which has been proven to be a required component to achieve development of a syncytium in host tissues [40]. Although we identified twelve genes predicted to encode for SPRY (SP1a and RYanodine receptor) domains, these putative SPRY-containing proteins lack a secretion signal, which is an essential requirement for the protein to be classified as a SPRYSEC ‘effector’. The presence of proteins with SPRY domains has been confirmed in the *R*. *similis* transcriptome analysis as well [41]. Within PPNs, proteins with SPRY domains and a secretion signal (SPRYSECs) have only been found in cyst nematodes and have been shown to be involved in suppressing and eliciting defense response in the host plants[42]. This absence of SPRYSEC effectors is consistent with *R*. *similis* not forming elaborate feeding sites in roots. However, effectors associated with host defense induction such as venom allergen- like effector proteins (VAPs) and suppression such as calreticulin (3) were present in *R*. *similis* [43,44] and one of these predicted calreticulins also possesses an ER-retention signals. Within PPNs, calreticulin has been extracted from the gland secretions of the root-knot nematode, *M*. *incognita* and the migratory PPN, *B*. *xylophilus*, where it has been shown to be involved in host defense suppression and reproduction respectively [43,45]. Homologues of genes involved in protection against host-derived oxidative stresses such as superoxide dismutase, peroxiredoxin, glutathione-S-transferase and glutathione-peroxidase were also found in *R*. *similis* (Table 1).Homologues of the S-phase kinase-associated protein, involved in ubiquitination pathways as well as the *FAR-1*/*SEC-2*, involved in the circumvention of host defense were also identified in *R*. *similis* [46,47]. *R*. *similis* also possesses a gene coding for a putative secreted chorismate mutase (with ~51% similarity), which is a key enzyme involved in modification of the host salicylic acid pathway [48]. A variety of proteases found in a broad spectrum of nematodes (irrespective of lifestyle) such as serine carboxypeptidase, cathepsin S, B and L were also identified in *R*. *similis* (Table B in S1 File). No homologues of the putative apoplastic effector 4D06 or the putative parasitic stage effector 30C02 or the plant peptide hormone mimic C-terminally Encoded Peptide (CEP) was found in the *R*. *similis* predicted protein set, further underscoring the lifestyle difference between a sedentary and a migratory PPN (see Table 1).

**Table 1:** Blast analysis of some known parasitism genes against *R*. *similis* predicted protein set.

Acc_No.	Gene_Name	# of hits	E-value	Similarity mean (%)
AHW98772.1	D406 protein [*Globodera rostochiensis*]	-	-	-
EN69461.1	30C02 [*Meloidogyne incognita*]	-	-	-
AHW98771.1	Peroxiredoxin [*Globodera rostochiensis*]	10	2.93E-126	65.27
2MFM_A	CEP [*Meloidogyne hapla*]	-	-	-
CAM84513.1	transthyretin-like protein 4 precursor, partial	10	1.56E-113	68.41
AHW98763.1	VAP1 protein [*Globodera rostochiensis*]	10	1.01E-80	59.57
AHW98759.1	CLAVATA3/ESR-related protein [*Globodera rostochiensis*]	-	-	-
AAR35032.1	SXP/RAL-2 protein [*Meloidogyne incognita*]	2	1.77E-25	54.76
AFK76483.1	Calreticulin	3	0	79.18
AHX24644.1	manganese superoxide dismutase, partial [Meloidogyne hapla]	6	3.32E-50	60.69
AAS82581.1	Chorismate mutase [*Meloidogyne incognita*]	1	8.08E-13	51.18
AAZ29194.1	Superoxide dismutase [*Meloidogyne incognita*]	6	2.75E-40	62.27
ABN64198.1	Glutathione-S-transferase [*Meloidogyne incognita*]	10	4.23E-71	60.62
Q06JG6.1	16D10 [*Meloidogyne javanica*]	-	-	-
CAA70477.2	Fatty acid and retinol-binding protein (FAR)[*G*. *pallida*]	3	1.92E-82	60.13

### Carbohydrate-Active enzymes (CAZymes)

CAZymes comprise of enzyme families that are involved in the degradation, synthesis and/or remodeling of carbohydrates and their conjugates. CAZymes were searched in the CAZy database, dbCAN database and the peptide pattern recognition (PPR) library to look for homologues of proteins encoding putative CAZymes in the predicted protein set of *R*. *similis*. Generally, CAZymes are divided into five broad classes based on their mode of action namely glycosyl hydrolase (GH), glycosyl transferase (GT), carbohydrate esterase (CE), polysaccharide lyase (PL) and auxillary activity (AA). GHs are enzymes that accelerate the degradation of glycosidic bond between carbohydrates and/or non-carbohydrates. GHs are found across a wide gamut of organisms including bacteria, plants, fungi and nematodes [49]. Within PPNs, GHs perform a range of functions, primarily, breakdown of plant cell walls which is made up of cellulose, xylans, arabinans and pectins, which can aid in increased movement through host cells. Additionally, GHs are also involved in the breakdown of simple and complex carbohydrates such as glucose and sucrose, which can be utilized as potential sources of nutrition by a PPN. The putative GHs in *R*. *similis* belong to multiple families, notably, GH5 (cellulase), and GH28 (polygalactouranase) (Table A in S1 File). In the *R*. *similis* genome, certain GHs such as GH5(cellulase) have carbohydrate binding modules (CBM) such as CBM2 appended. Cellulases from the root-knot nematode *M*. *incognita* and the cyst nematode, *G*. *pallida* also possess an appended CBM2 domain. In the sugar beet cyst nematode, *H*. *schachtii*, a secreted protein with a CBM domain, was demonstrated to directly interact with an *Arabidopsis* pectin methylesterase, thereby implicating it in facilitating nematode parasitism [50].

**Fig 1:**
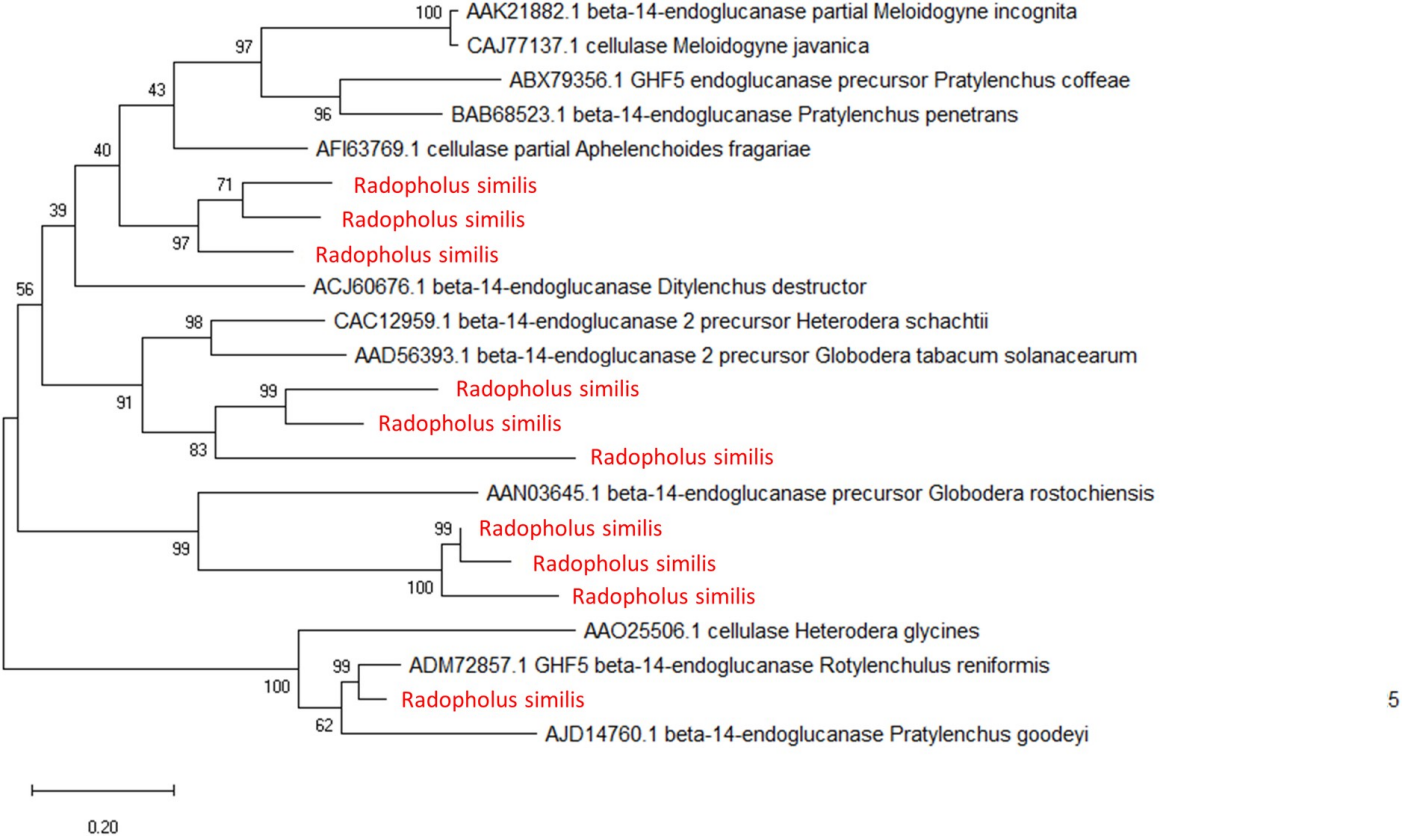
Evolutionary analysis of cellulase genes. Phylogenetic analysis based on concatenated alignments of 22 cellulase protein sequences from multiple plant-parasitic nematode in clade 10 and clade 12. The evolutionary history was inferred by using the maximum-likelihood method with the best-fit (WAG+G+I) substitution model predicted using MEGA X. Numbers indicate bootstrap values. 1000 bootstrap replications were performed. *R*. *similis* cellulase genes are highlighted in red. Evolutionary analysis was performed using MEGA X.

*R*. *similis* encodes ten putative cellulase genes belonging to the GH5 family, subfamily 2 Phylogenetic analyses demonstrated that the *R*. *similis* GH5 proteins are distributed in four distinct clades, of which three are clustered and each is closely related to the cyst nematodes belonging to the *Heterodera* and *Globodera* genus (Fig 1). This result supports the previous SSU rDNA studies, which determined that *R*. *similis* is evolutionarily closer to cyst nematodes than to root knot nematodes [24]. Moreover, in one of the clades we observed that *R*. *similis* shares a sister relationship with *Rotylenchulus reniformis*, a semi-endoparasitic nematode that serves as a potential intermediary between the migratory parasite, *R*. *similis* and the sedentary parasite, *H*. *glycines*. Multiple effector studies and a recent phylogenetic study by Holterman et al. [32] have supported a common ancestry hypothesis between the semi-endoparasitic reniform nematode belonging to the Rotylenchulidae family and the endoparasitic cyst nematodes of the Heteroderidae family [32,51]. This particular observation also corroborates previous findings as well as the hypothesis put forth by van Megen et al. [32,52] that “members of the genus *Radopholus* could be relatively closely related to the common ancestor of the Hoplolaimidae and Heteroderidae”[24,32,52]. It can also be noted that three *R*. *similis* proteins shows possible sister relationships with other PPNs besides the cyst nematodes. However, the bootstrap support value is less than 50% and hence its’ not considered as a significant association. GH5 cellulases have been shown to be conserved in diverse clade 12 nematodes including the root-knot and cyst nematodes, the family Pratylenchidae and the genus *Radopholus* and have been hypothesized to be acquired from a common ancestral gene via HGT from bacteria [53]. Additionally, we analyzed the presence/absence of N-terminal signal peptides in these putative cellulases and found that nine out of the ten cellulases possess a signal peptide with no transmembrane domain. The putative subcellular localization patterns of these cellulases were predicted based on protein sequence information using DeepLoc [54]. DeepLoc predicted nine of these ten putative cellulase proteins to be ‘soluble’ and to be ‘extracellularly’ secreted (Table C in S1 File). Following this, we compared the predicted localization pattern of this protein with the localization of a known cellulase gene from a PPN (in this case *H*. *glycines*) and found the predicted localization likelihood patterns to be extracellular (S3 Fig). No cellulases belonging to the GH45 family were predicted in the *R*. *similis* protein set during our analysis, supporting the evidence of independent origins (bacterial and fungal) of cellulases in most PPNs [53,55].

A single gene coding for a putative polygalactouranase, belonging to the GH28 family has also been found in *R*. *similis*. Polygalactouranase is required for the breakdown of galactouronic acid, which is an essential monomer that makes up pectin. GH28 polygalactouranase has also been found in the transcriptome of *R*. *similis* by Huang et al. [41]. A BLAST analysis of this gene against the NCBI nr database indicates that the sequence is homologous to the GH28 gene of the bacterial soil-borne pathogen *Ralstonia solanacearum*. Intriguingly, *R*. s*olanacearum* is a xylem colonising pathogen and *R*. *similis* has been reported to block xylem vessels which might imply that these functions might have been a characterisic of niche specialization. Furthermore, within plant-parasitic nematodes, GH28 genes acquired from bacteria have been found in root-knot nematode *M*. *hapla*, *M*. *incognita* and the family Pratylenchidae. Notably, polygalactouranase from RKNs as well as the false root-knot nematode, *Nacobbus aberrans* also demonstrates high sequence similarity with the *R*. *solanacearum* GH28 protein [9,31,56]. Significantly, the semi-endoparasitic nematode, *R*. *reniformis* also possesses GH28s, but the GH28 in *R*. *reniformis* appears to be non-functional. Because GH28 does appear to be functional in *R*. *similis*, this finding supports the hypothesis of early gain in the last common ancestor of root knot and cyst, and subsequent loss in the cyst nematodes [31].

Eight genes encoding for putative polysaccharide lyases (PL) have been predicted in *R*. *similis*. These genes belong to five families, namely PL1, PL3, PL5, PL9 and PL22. *R*. *similis* possesses four putative PL3 (pectate lyase) proteins, fewer than other PPNs in clade 12 such as *M*. *hapla* (20), *M*. *incognita* (33), *H*. *glycines* (15), *G*. *pallida* (8) and the clade 10 PPN *B*. *xylophilus* (15) (Table A in S1 File). Pectate lyase catalyze the breakdown and degradation of pectins, which is an important component of plant cell wall, in addition to hemicellulose and cellulose [57]. In addition to cellulase, pectate lyase induce softening of plant tissues, which in turn aids in movement and feeding of the nematode. Pectate lyases along with cellulases and a host of other secretory proteins are released into the plant cell during infection and feeding by the nematode. SignalP was used to search for signal peptides in these PL3 sequences in order to categorize these proteins as ‘secretory’. We found that only three of the putative PL3 proteins show presence of conserved signal peptide sequences in their N-terminal, (Table D in S1 File) and they do not possess a transmembrane domain. Pectate lyases have also been used to study phylogenetic associations in sedentary and migratory endoparasitic nematodes [58,59]. We performed phylogenetic analysis on the four PL3s and found a pattern similar to the GH5 cellulase phylogenetic tree (S1 Fig). However, the putative PL3s received a lower bootstrap support value when compared with most GH5 cellulases. The *R*. *similis* PL3s are clustered into a monophyletic clade and share a common ancestor with the cyst nematodes belonging to the *Globodera* and *Heterodera* genus Additionally, the branch length leading to the cyst nematodes from the common ancestor is relatively longer when compared with the branch length leading to the *R*. *similis* PL3s. This might indicate higher divergence and increased substitutions per site relative to the ancestor as well as *R*. *similis*.

### Phylogenetic analysis of orthologous genes

**Fig 2:**
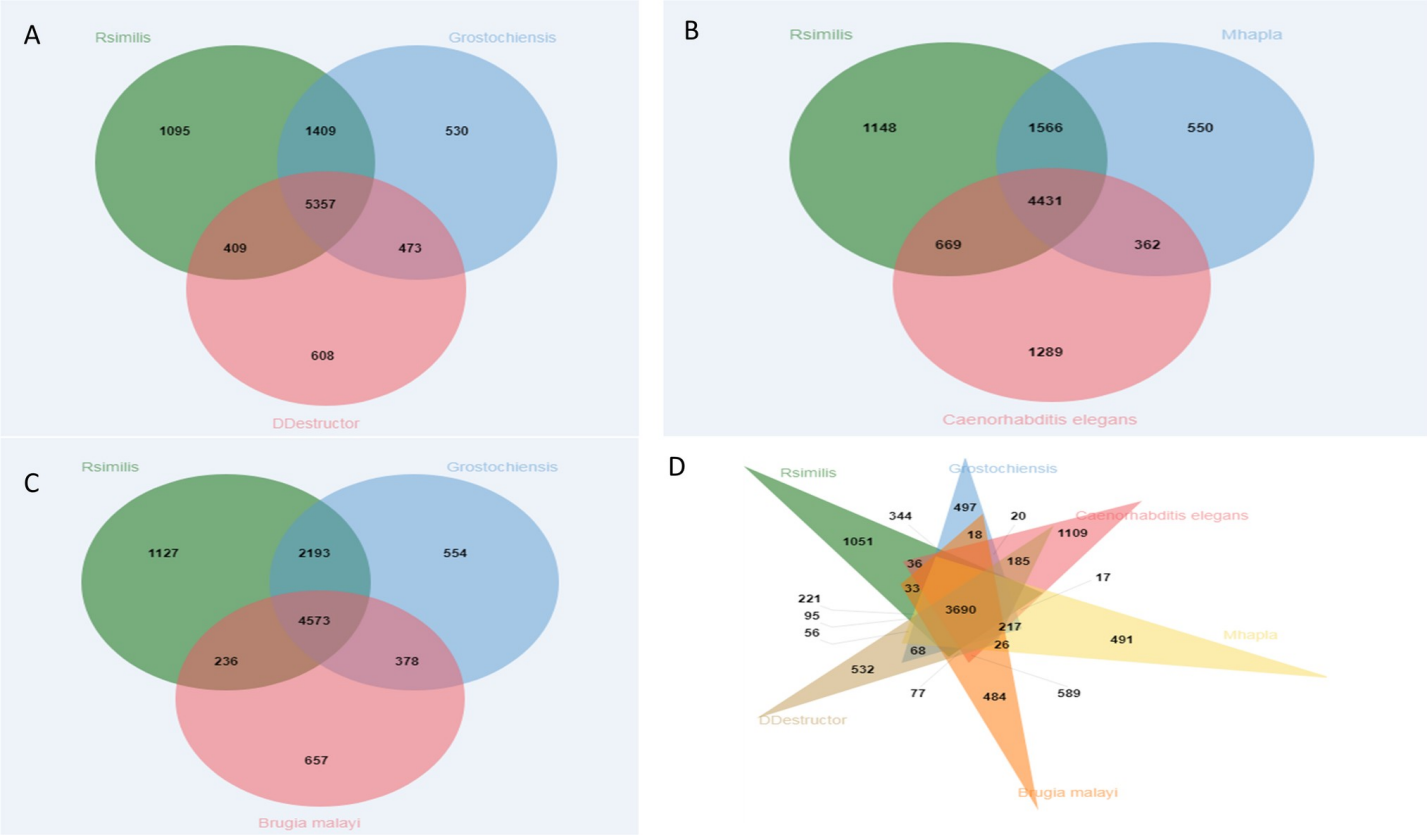
Venn diagrams displaying overlapping ortholog clusters shared by *R*. *similis*. (**A**) Orthologous clusters shared between *R*. *similis*, the potato cyst nematode *G*. *rostochiensis* and the potato rot nematode *D*. *destructor* (**B**) Orthologous clusters shared between *R*. *similis*, the root-knot nematode *M*. *hapla* and the free-living nematode *C*. *elegans* (**C**) Orthologous clusters shared between *R*. *similis*, the potato cyst nematode *G*. *rostochiensis* and the human parasitic filarial nematode *B*. *malayi* (**D**) Orthologous clusters shared between *R*. *similis*, *G*. *rostochiensis*, *M*. *hapla*, *C*. *elegans*, *D*. *destructor* and *B*. *malayi*.

Plant-parasitic nematodes occur in clade 1 (Triplonchida), clade 2 (Dorylaimida), clade 10 (Aphelencoididae) and clade 12 (Tylenchida) of the 12 clades comprising the phylum Nematoda. In a phylogenetic tree presented by Holterman et al., 2017 [32], disparate gene loss and gain events in parasitic abilities are seen among nematode lineages. We identified orthologous gene clusters shared by *R*. *similis* with other nematodes such as plant-parasitic, human-parasitic and free-living. *R*. *similis* forms a total of 8542 orthologous clusters and 5115 singletons. All the species combined, shared a total of 3690 orthologous clusters (Fig 2). We observed that *R*. *similis* shares higher number of orthologous clusters with the cyst nematode *G*. *rostochiensis* (4034), compared to the root-knot nematode *M*. *hapla* (3911), the free-living nematode *C*. *elegans* (3726), the migratory endoparasitic nematode *D*. *destructor* (3785) and the human parasitic nematode *B*. *malayi* (3723).

**Fig 3:**
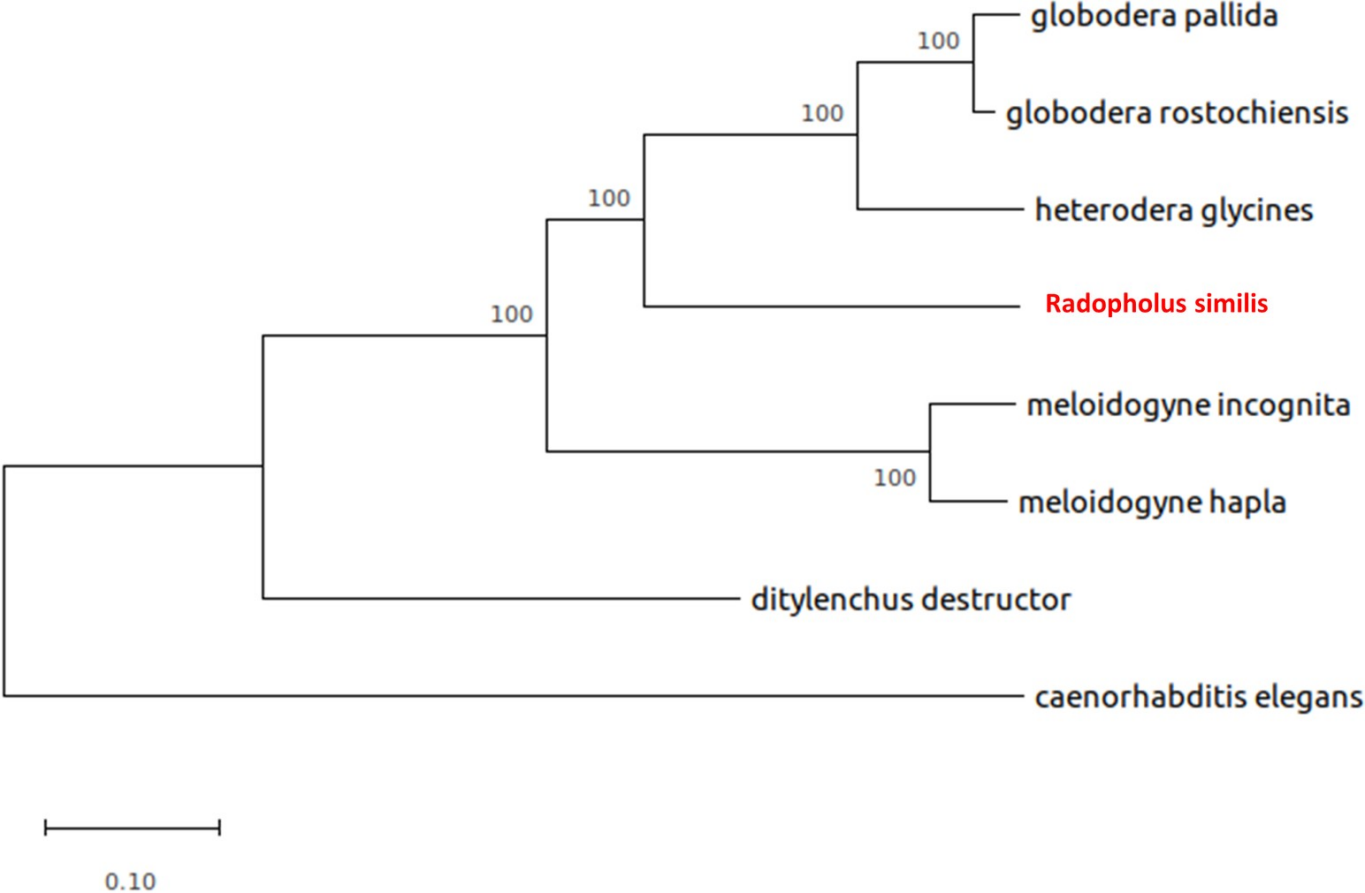
Characterization of species phylogenetic tree. Species tree constructed using concatenated alignments of 1630 orthogroups with minimum of 75% of species having single-copy genes in any orthogroup using the LG + G substitution model determined by MEGA X. Numbers on the branch length indicates bootstrap support value. Bootstrap values were calculated from 1000 replicates. Branch length indicates the number of substitutions per site.

Additionally, alignments of 1630 orthologs for eight different species specifically *R*. *similis*, *G*. *pallida*, *G*. *rostochiensis*, *H*. *glycines*, *M*. *incognita M*. *hapla*, *D*. *destructor and C*. *elegans* was performed to gain insight into the phylogenetic relationships shared by *R*. *similis*. The resulting species tree corroborates the existing hypothesis, that, *R*. *similis* is indeed a sister taxon to the cyst nematodes (*G*. *rostochiensis*, *G*. *pallida* and *H*. *glycines*) and thereby share the latest common ancestor with the cyst nematodes than with the root-knot nematodes, *M*. *hapla* and *M*. *incognita* or the migratory endoparasite *D*. *destructor* (Fig 3). [24,60].

## Conclusion

Our study confirms the close association between *R*. *similis* and the cyst nematodes specifically that *R*. *similis* and the cyst nematodes share the latest common ancestor. In multiple analysis that we have performed, including phylogenetic analysis of single-copy orthologs, analysis of secreted proteins such as cellulase, pectate lyase as well as the housekeeping *gpd* gene, *R*. *similis* appears as a sister taxon of the cyst nematodes (see Fig 1, Fig 2, S2 Fig and S3 Fig), reinforcing previous findings [24]. Additionally, our data conforms well with the transcriptome analysis by Huang et al. [41], specifically, the absence of putative cyst nematode genes that code for CLE-like peptides and the presence of the GH28 gene as a significant indicator. Our findings reveal a basic set of effectors for a non-feeding site forming migratory endoparasitic nematode and further support early evolutionary relationships to the Hoplolaimidae and Heteroderidae.

Transitioning from a migratory lifestyle to a sedentary one has been a giant leap in the evolution of plant-parasitic nematodes and an important milestone in achieving an intimate host-parasite interaction. Sedentary endoparasitism has been proposed to have evolved independently through five separate pathways in clade 12 of the phylum Nematoda [32]. Additionally, even within the sedentary endoparasitic nematodes, the cyst and root-knots, have few parallels in their feeding site development or their effector suite, indicating further disparate origins [61]. When this divergence occurred in the evolutionary transition is unclear. Perhaps the more intriguing question is why and what underlying evolutionary forces were in play that compelled a PPN to transition from a migratory to a sedentary lifestyle or possess distinct arsenals for establishing a sedentary lifestyle. Future work which analyzes *R*. *similis* genomes and the genomes of numerous sedentary endoparasitic nematodes from different geographical locations and ecological niches can provide further insight into these evolutionary patterns. Additionally, functional characterization studies of key effectors common between *R*. *similis*, the cyst nematodes and the semi-endoparasitic *R*. *reniformis*, could provide tell-tale signatures regarding the evolution of endoparasitism in plant-parasitic nematodes. With these data, we hope to further the understanding of the complex multipartite parasitism mechanisms that makes *R*. *similis* an economically important pathogen.

## Materials and methods

### Gene prediction

Gene prediction was performed using the MAKER v.2.31.8 pipeline [62] in three rounds. For the gene predictions, 7,382 *R*. *similis* ESTs were extracted from the NCBI EST database, following which the ESTs were used as evidence to make gene predictions for the genome along with an in-house built protein set constructed from the proteins of closely related nematodes downloaded from wormbase parasite and proteins from the uniprot-swissprot database. These evidence-based predictions were then integrated with ab-initio predictions performed by SNAP and AUGUSTUS to obtain a more robust and complete set of gene predictions. Finally, the gene predictions with Annotation edit distance (AED) values less than 1 were extracted using a custom perl script.

### CAZyme

*R*. *similis* protein sequences were blasted against CAZy database [49] (using Diamond), dbCAN database (using HMMER) and Peptide pattern recognition (PPR) (using hotpep) to search for putative CAZymes in the *R*. *similis* proteome (34,55). The E value used was 1e-5. dbCAN2 was utilized to perform this job[63] Hits returned in at least two databases were considered as valid candidates. For annotation of the CBM moieties attached with the GHs, hits returned from DIAMOND were considered. Data for the other nematodes were from [64]. However, the genome papers of the corresponding nematodes and pertinent literature was also reviewed to ensure accuracy while reporting.

For annotating the expansins in *R*. *similis* predicted protein set, known expansin proteins sequences of plant-parasitic nematodes were downloaded from NCBI. Following this, the downloaded protein dataset was used to create a database for Blastp. *Radopholus* predicted protein set was used as query against this database. The E-value used was 1e-5. The data for expansins for the other nematodes were taken from [41].

### Phylogenetic analysis of secreted proteins and gpd

A position specific iterative (PSI) blastp analysis of relevant genes was done against the NCBI nr database (nematoda) with an E- value of 1e-5. A blastp analysis was also performed on wormbase parasite with an e-value of 1e-5. The relevant hits were extracted, following which the sequences were aligned using MUSCLE [65] (Gap open penalty -2.90, Gap extend penalty 0.00 and hydrophobicity multiplier1.20). The cluster method selected was UPGMA. The aligned sequences were then concatenated and utilized to construct a tree using the maximum likelihood statistical method using the best-fit substitution model estimated using MEGA X [66]. Site coverage was set to be 95% with partial deletion of gaps/missing data. Resampling was performed using bootstrap analysis of 1000 replicates. For the gpd gene, *C*. *elegans* gpd genes were used as query to conduct a blastp against the *R*. *similis* protein database. Genes that returned hits with an E-value = 0.0 were used to query NCBI or wormbase.

### Signal peptide and localization pattern prediction

Signal peptides and transmembrane domains in the protein sequences were predicted using SignalP4.1 [67]. The localization pattern of proteins sequences were predicted using the DeepLoc server [54] with the ‘Eukaryote’ option selected.

### Phylogenetic analysis using orthologs

For constructing the venn diagram OrthoMCL [68] was utilized to identify orthologous clusters. An all against all blastp was performed with an E value of 1e-5 and the inflation value of 1.5 to balance sensitivity and selectivity of clusters. Orthovenn [69] was utilized to construct venn diagrams using orthologous clusters. Genome and protein sequences for other nematodes were downloaded from wormbase parasite [70,71].

For constructing the species tree, protein sequences of *R*. *similis* and eight other nematodes such as *Globodera pallida*, *Globodera rostochiensis*, *Heterodera glycines*, *Meloidogyne incognita*, *Meloidogyne hapla*, *Ditylenchus destructor* and *Caenorhabditis* elegans were analyzed by OrthoFinder [72]. Alignments was generated using MUSCLE [65] in Orthofinder. The MUSCLE generated alignments were concatenated and the species tree was constructed X[66] with the Maximum-likelihood statistical best fit method (LG+G model) estimated using MEGA X with 1000 bootstrap replications. All positions with less than 95% site coverage were eliminated. That is, fewer than 5% alignment gaps, missing data, and ambiguous bases were allowed at any position … The free-living nematode *C*. *elegans* was used as an outgroup.

## NCBI accession number

The Whole genome shotgun (WGS) has been deposited in NCBI under the accession number SJFO00000000 and BioProject PRJNA522283.

## Supporting information

S1 File (DOCX)

S1 Fig Phylogenetic analysis of *R*. *similis* pectate lyase 3. *R*. *similis* proteins are indicated in red. Genbank accession numbers are shown adjacent to each nematode. The numbers on the branches indicate bootstrap support value. 1000 bootstrap replications were performed. Scale indicates number of substitutions per site.
(TIF)

S2 Fig Phylogenetic analysis of *R*. *similis* gpd gene. *R*. *similis* gpd gene is indicated in red. The numbers on the branches indicate bootstrap support value. 1000 bootstrap replications were performed. Scale indicates number of substitutions per site.
(TIF)

S3 Fig Localization pattern of cellulase sequences. (A) *R*. *similis* predicted cellulase protein sequence and (B) *H*. *glycines* cellulase protein sequence (acc: AAC15707.1). Numbers on branches indicate localization likelihood in different compartments. Ideal localization path is highlighted in red.
(TIF)
