# Supplementary material for: The genome of the migratory nematode, Radopholus similis, reveals signatures of close association to the sedentary cyst nematodes
Source: PLoS One. 2019 Oct 25;14(10):e0224391. doi: 10.1371/journal.pone.0224391 (PMC6814228; doi:10.1371/journal.pone.0224391)
Supplement: S1 File — (DOCX) [file pone.0224391.s001.docx]

**Supporting information**

**Table A. CAZyme repertoire of *R. similis* compared to other nematodes**

| **CAZy Family** | **Rs** | **Bx** | **Ce** | **Gp** | **Me** | **Mh** | **Mi** | **Gr** | **Hg** |
| --- | --- | --- | --- | --- | --- | --- | --- | --- | --- |
| GH104 | 0 | 0 | 0 | 2 | 0 | 0 | 0 | 0 | 0 |
| GH20 | 5 | 5 | 5 | 2 | 2 | 3 | 4 | 3 | 6 |
| GH22 | 0 | 0 | 0 | 0 | 0 | 0 | 1 | 0 | 0 |
| GH25 | 0 | 3 | 10 | 1 | 10 | 5 | 5 | 0 | 0 |
| GH27 | 0 | 3 | 1 | 0 | 1 | 1 | 1 | 1 | 2 |
| GH28 | 1 | 0 | 0 | 0 | 0 | 4 | 4 | 0 | 0 |
| GH29 | 1 | 3 | 1 | 0 | 1 | 1 | 2 | 1 | 2 |
| GH3 | 6 | 0 | 0 | 1 | 0 | 0 | 0 | 0 | 0 |
| GH30 | 5 | 9 | 4 | 0 | 5 | 4 | 8 | 0 | 0 |
| GH45 | 0 | 11 | 0 | 0 | 0 | 0 | 0 | 0 | 0 |
| GH5 | 10 | 0 | 0 | 15 | 8 | 10 | 31 | 6 | 8 |
| GH53 | 4 | 0 | 0 | 1 | 0 | 0 | 0 | 1 | 1 |
| GH56 | 0 | 3 | 1 | 0 | 0 | 1 | 2 | 0 | 0 |
| GH59 | 0 | 0 | 1 | 0 | 0 | 0 | 0 | 0 | 0 |
| GH75 | 0 | 0 | 1 | 0 | 2 | 1 | 1 | 0 | 0 |
| GH76 | 0 | 0 | 1 | 0 | 0 | 1 | 0 | 0 | 0 |
| GH82 | 0 | 0 | 0 | 0 | 0 | 1 | 0 | 0 | 0 |
| GH84 | 1 | 1 | 1 | 1 | 1 | 1 | 1 | 1 | 1 |
| GH85 | 1 | 1 | 1 | 0 | 1 | 1 | 1 | 1 | 0 |
| GH89 | 0 | 0 | 1 | 0 | 0 | 0 | 0 | 0 | 0 |
| GH9 | 0 | 1 | 0 | 0 | 0 | 0 | 0 | 0 | 0 |
| GH99 | 1 | 2 | 0 | 1 | 0 | 0 | 0 | 1 | 2 |
| GH15 | 1 | 2 | 2 | 0 | 0 | 0 | 0 | 0 | 0 |
| GH16 | 4 | 8 | 0 | 0 | 0 | 0 | 0 | 0 | 0 |
| GH18 | 3 | 12 | 39 | 11 | 6 | 9 | 3 | 6 | 7 |
| GH19 | 2 | 2 | 4 | 2 | 0 | 1 | 4 | 2 | 7 |
| GH2 | 2 | 4 | 2 | 1 | 0 | 2 | 5 | 0 | 0 |
| PL22 | 1 | 1 | 1 | 0 | 0 | 0 | 0 | 0 | 0 |
| PL3 | 4 | 15 | 0 | 8 | 11 | 20 | 33 | 3 | 15 |

*data for *Bx, Ce, Gp, Me, Mh, Mi* from [1]

**Table B. PPN effector protein sequences blasted with *R. similis***

| Blasted? | Accession No. | Gene description | no. of hits | E-value | Sim mean |
| --- | --- | --- | --- | --- | --- |
| [BLASTED] | AJR19784.1 | esophageal gland-localized secretory protein 16 [Heterodera glycines] | 2 | 1.12E-12 | 52.75 |
| [BLASTED] | AJR19771.1 | esophageal gland-localized secretory protein 3 [Heterodera glycines] | 1 | 4.98E-33 | 77.23 |
| [BLASTED] | AVA09730.1 | putative effector protein [Heterodera avenae] | 5 | 2.61E-38 | 55.71 |
| [BLASTED] | AVA09729.1 | putative effector protein [Heterodera avenae] | 10 | 3.16E-152 | 51.1 |
| [BLASTED] | AVA09724.1 | putative effector protein [Heterodera avenae] | 10 | 1.51E-48 | 61.9 |
| [NO-BLAST] | AVA09723.1 | putative effector protein [Heterodera avenae] |  |  |  |
| [BLASTED] | AVA09722.1 | putative effector protein [Heterodera avenae] | 10 | 7.56E-64 | 66.4 |
| [BLASTED] | AVA09716.1 | putative effector protein [Heterodera avenae] | 1 | 3.37E-09 | 58.23 |
| [BLASTED] | AVA09713.1 | putative effector protein [Heterodera avenae] | 7 | 7.85E-70 | 58.61 |
| [BLASTED] | AVA09712.1 | putative effector protein [Heterodera avenae] | 10 | 2.37E-75 | 65.11 |
| [NO-BLAST] | AVA09711.1 | putative effector protein [Heterodera avenae] |  |  |  |
| [BLASTED] | AVA09710.1 | putative effector protein [Heterodera avenae] | 10 | 9.22E-44 | 61.77 |
| [BLASTED] | AVA09709.1 | putative effector protein [Heterodera avenae] | 7 | 3.65E-50 | 50.63 |
| [BLASTED] | AVA09708.1 | putative effector protein [Heterodera avenae] | 1 | 2.23E-79 | 85.23 |
| [NO-BLAST] | AVA09707.1 | putative effector protein [Heterodera avenae] |  |  |  |
| [BLASTED] | AVA09706.1 | putative effector protein [Heterodera avenae] | 10 | 5.01E-56 | 65.33 |
| [BLASTED] | AVA09705.1 | putative effector protein [Heterodera avenae] | 10 | 6.43E-28 | 50.49 |
| [BLASTED] | AVA09704.1 | putative effector protein [Heterodera avenae] | 3 | 7.82E-08 | 52.02 |
| [BLASTED] | AVA09703.1 | putative effector protein [Heterodera avenae] | 10 | 5.97E-67 | 48.11 |
| [BLASTED] | AVA09702.1 | putative effector protein [Heterodera avenae] | 9 | 1.68E-37 | 49.75 |
| [NO-BLAST] | AVA09701.1 | putative effector protein [Heterodera avenae] |  |  |  |
| [BLASTED] | AVA09698.1 | putative effector protein [Heterodera avenae] | 10 | 1.29E-122 | 68.94 |
| [BLASTED] | AVA09697.1 | putative effector protein [Heterodera avenae] | 2 | 6.32E-18 | 60.1 |
| [BLASTED] | AVA09696.1 | putative effector protein [Heterodera avenae] | 8 | 2.11E-49 | 58.69 |
| [NO-BLAST] | AVA09695.1 | putative effector protein [Heterodera avenae] |  |  |  |
| [BLASTED] | AVA09694.1 | putative effector protein [Heterodera avenae] | 5 | 2.33E-15 | 51.55 |
| [BLASTED] | AVA09693.1 | putative effector protein [Heterodera avenae] | 3 | 2.16E-32 | 58.92 |
| [NO-BLAST] | AVA09692.1 | putative effector protein [Heterodera avenae] |  |  |  |
| [BLASTED] | AVA09691.1 | putative effector protein [Heterodera avenae] | 1 | 1.22E-118 | 62.61 |
| [BLASTED] | AVA09690.1 | putative effector protein [Heterodera avenae] | 7 | 4.71E-162 | 86.75 |
| [BLASTED] | AVA09689.1 | putative effector protein [Heterodera avenae] | 10 | 2.27E-108 | 57.14 |
| [BLASTED] | AVA09688.1 | putative effector protein [Heterodera avenae] | 10 | 2.61E-94 | 60.82 |
| [BLASTED] | AVA09687.1 | putative effector protein [Heterodera avenae] | 10 | 3.16E-152 | 51.1 |
| [BLASTED] | AVA09686.1 | putative effector protein [Heterodera avenae] | 1 | 2.51E-28 | 69 |
| [BLASTED] | AVA09685.1 | putative effector protein [Heterodera avenae] | 1 | 2.75E-23 | 39.46 |
| [BLASTED] | AVA09684.1 | putative effector protein [Heterodera avenae] | 1 | 9.87E-12 | 43.1 |
| [BLASTED] | AVA09683.1 | putative effector protein [Heterodera avenae] | 10 | 6.34E-139 | 57.22 |
| [BLASTED] | AVA09682.1 | putative effector protein [Heterodera avenae] | 10 | 2.00E-157 | 65.23 |
| [BLASTED] | AVA09681.1 | putative effector protein [Heterodera avenae] | 7 | 8.99E-161 | 87.07 |
| [BLASTED] | AVA09680.1 | putative effector protein [Heterodera avenae] | 2 | 8.23E-77 | 79.02 |
| [BLASTED] | AVA09679.1 | putative effector protein [Heterodera avenae] | 2 | 0 | 70.4 |
| [BLASTED] | AVA09678.1 | putative effector protein [Heterodera avenae] | 1 | 5.02E-41 | 58.82 |
| [BLASTED] | AVA09677.1 | putative effector protein [Heterodera avenae] | 3 | 3.47E-26 | 52.55 |
| [BLASTED] | AVA09676.1 | putative effector protein [Heterodera avenae] | 1 | 1.80E-24 | 47.09 |
| [BLASTED] | AVA09675.1 | putative effector protein [Heterodera avenae] | 10 | 0 | 54.23 |
| [BLASTED] | AVA09674.1 | putative effector protein [Heterodera avenae] | 1 | 5.67E-19 | 62.75 |
| [BLASTED] | AVA09673.1 | putative effector protein [Heterodera avenae] | 4 | 0 | 51.14 |
| [NO-BLAST] | AVA09672.1 | putative effector protein [Heterodera avenae] |  |  |  |
| [BLASTED] | AVA09671.1 | putative effector protein [Heterodera avenae] | 10 | 3.54E-169 | 55.38 |
| [BLASTED] | AVA09670.1 | putative effector protein [Heterodera avenae] | 10 | 0 | 59.01 |
| [BLASTED] | AVA09669.1 | putative effector protein [Heterodera avenae] | 10 | 2.37E-75 | 65.11 |
| [BLASTED] | AVA09668.1 | putative effector protein [Heterodera avenae] | 1 | 1.85E-51 | 89.01 |
| [BLASTED] | AVA09667.1 | putative effector protein [Heterodera avenae] | 10 | 6.61E-48 | 64.31 |
| [BLASTED] | AVA09666.1 | putative effector protein [Heterodera avenae] | 5 | 2.48E-57 | 61.23 |
| [NO-BLAST] | AVA09665.1 | putative effector protein [Heterodera avenae] |  |  |  |
| [BLASTED] | AVA09664.1 | putative effector protein [Heterodera avenae] | 3 | 1.81E-87 | 53.9 |
| [BLASTED] | AVA09663.1 | putative effector protein [Heterodera avenae] | 1 | 1.37E-98 | 66.67 |
| [BLASTED] | AVA09662.1 | putative effector protein [Heterodera avenae] | 10 | 9.73E-122 | 56.17 |
| [BLASTED] | AVA09661.1 | putative effector protein [Heterodera avenae] | 8 | 1.62E-60 | 45.43 |
| [BLASTED] | AVA09660.1 | putative effector protein [Heterodera avenae] | 10 | 5.97E-67 | 48.11 |
| [BLASTED] | AVA09659.1 | putative effector protein [Heterodera avenae] | 8 | 0 | 52.56 |
| [BLASTED] | AVA09658.1 | putative effector protein [Heterodera avenae] | 4 | 2.27E-54 | 57.21 |
| [BLASTED] | AVA09657.1 | putative effector protein [Heterodera avenae] | 9 | 7.90E-53 | 54.48 |
| [BLASTED] | AVA09656.1 | putative effector protein [Heterodera avenae] | 10 | 1.91E-129 | 49.73 |
| [BLASTED] | AVA09655.1 | putative effector protein [Heterodera avenae] | 5 | 0 | 58.84 |
| [BLASTED] | AVA09654.1 | putative effector protein [Heterodera avenae] | 5 | 1.15E-51 | 47.09 |
| [BLASTED] | AVA09653.1 | putative effector protein [Heterodera avenae] | 10 | 0 | 55.82 |
| [BLASTED] | AVA09652.1 | putative effector protein [Heterodera avenae] | 1 | 1.22E-118 | 62.61 |
| [BLASTED] | AVA09651.1 | putative effector protein [Heterodera avenae] | 3 | 9.30E-44 | 60.81 |
| [BLASTED] | AVA09650.1 | putative effector protein [Heterodera avenae] | 10 | 0 | 58.23 |
| [BLASTED] | AVA09649.1 | putative effector protein [Heterodera avenae] | 10 | 0 | 63.47 |
| [BLASTED] | AVA09648.1 | putative effector protein [Heterodera avenae] | 10 | 4.34E-106 | 59.9 |
| [BLASTED] | AVA09647.1 | putative effector protein [Heterodera avenae] | 10 | 3.82E-101 | 59.19 |
| [BLASTED] | AVA09646.1 | putative effector protein [Heterodera avenae] | 3 | 1.58E-149 | 60.4 |
| [BLASTED] | AVA09645.1 | putative effector protein [Heterodera avenae] | 3 | 7.63E-141 | 61.52 |
| [BLASTED] | AVA09644.1 | putative effector protein [Heterodera avenae] | 2 | 3.17E-56 | 48.76 |
| [BLASTED] | AVA09643.1 | putative effector protein [Heterodera avenae] | 1 | 1.16E-38 | 64.74 |
| [BLASTED] | AVA09642.1 | putative effector protein [Heterodera avenae] | 2 | 1.67E-59 | 58.97 |
| [BLASTED] | AVA09641.1 | putative effector protein [Heterodera avenae] | 2 | 8.61E-60 | 59.41 |
| [BLASTED] | AVA09640.1 | putative effector protein [Heterodera avenae] | 5 | 4.48E-19 | 49.77 |
| [NO-BLAST] | AVA09639.1 | putative effector protein [Heterodera avenae] |  |  |  |
| [BLASTED] | AVA09638.1 | putative effector protein [Heterodera avenae] | 2 | 3.93E-149 | 77.96 |
| [BLASTED] | AVA09637.1 | putative effector protein [Heterodera avenae] | 10 | 7.92E-116 | 66.46 |
| [NO-BLAST] | AJR19786.1 | esophageal gland-localized secretory protein 18 [Heterodera glycines] |  |  |  |
| [NO-BLAST] | AJR19785.1 | esophageal gland-localized secretory protein 17 [Heterodera glycines] |  |  |  |
| [BLASTED] | AJR19782.1 | esophageal gland-localized secretory protein 14 [Heterodera glycines] | 3 | 2.94E-63 | 66.13 |
| [BLASTED] | AJR19781.1 | esophageal gland-localized secretory protein 13 [Heterodera glycines] | 9 | 3.63E-53 | 49.77 |
| [BLASTED] | AJR19780.1 | esophageal gland-localized secretory protein 12 [Heterodera glycines] | 6 | 1.15E-50 | 59.29 |
| [NO-BLAST] | AJR19779.1 | esophageal gland-localized secretory protein 11 [Heterodera glycines] |  |  |  |
| [NO-BLAST] | AJR19777.1 | esophageal gland-localized secretory protein 9 [Heterodera glycines] |  |  |  |
| [NO-BLAST] | AJR19776.1 | esophageal gland-localized secretory protein 8 [Heterodera glycines] |  |  |  |
| [NO-BLAST] | AJR19772.1 | esophageal gland-localized secretory protein 4 [Heterodera glycines] |  |  |  |
| [NO-BLAST] | AJR19770.1 | esophageal gland-localized secretory protein 2 [Heterodera glycines] |  |  |  |
| [NO-BLAST] | AJR19769.1 | esophageal gland-localized secretory protein 1 [Heterodera glycines] |  |  |  |
| [BLASTED] | AHX24637.1 | beta-1,4-endoglucanase-1, partial [Meloidogyne hapla] | 10 | 5.95E-27 | 70.48 |
| [BLASTED] | AHX24632.1 | calreticulin, partial [Meloidogyne hapla] | 3 | 7.49E-98 | 77.39 |
| [BLASTED] | AHX24628.1 | cathepsin L protease, partial [Meloidogyne hapla] | 10 | 1.21E-33 | 62.19 |
| [NO-BLAST] | AHA80140.1 | CLAVATA3/ESR-related protein [Globodera tabacum ssp. 'azteca'] |  |  |  |
| [NO-BLAST] | AHA80124.1 | CLAVATA3/ESR-related protein [Globodera virginiae] |  |  |  |
| [NO-BLAST] | AHB30308.1 | CLAVATA3/ESR-related protein [Globodera virginiae] |  |  |  |
| [BLASTED] | AHZ59334.1 | truncated secreted SPRY domain-containing protein 15, partial [Globodera rostochiensis] | 2 | 4.03E-29 | 58.57 |
| [BLASTED] | AHW98770.1 | S-phase kinase-associated protein 1 [Globodera rostochiensis] | 5 | 1.16E-113 | 58.53 |
| [BLASTED] | AHW98769.1 | secreted glutathione peroxidase [Globodera rostochiensis] | 10 | 3.35E-150 | 63.01 |
| [BLASTED] | AHW98768.1 | putative amphid protein [Globodera rostochiensis] | 2 | 2.08E-13 | 51.08 |
| [BLASTED] | AHW98767.1 | E9 protein, partial [Globodera rostochiensis] | 10 | 3.58E-148 | 63.1 |
| [BLASTED] | AHW98766.1 | matrix metalloproteinase [Globodera rostochiensis] | 7 | 3.59E-87 | 52.57 |
| [BLASTED] | AHW98765.1 | pectate lyase 2 precursor [Globodera rostochiensis] | 10 | 5.31E-65 | 55.15 |
| [BLASTED] | AHW98764.1 | Gr-pel1 pectate lyase 1 [Globodera rostochiensis] | 9 | 1.27E-41 | 52.56 |
| [BLASTED] | AHW98762.1 | beta-1,4-endoglucanase precursor [Globodera rostochiensis] | 10 | 1.89E-102 | 60.35 |
| [BLASTED] | AHW98761.1 | beta-1,4-endoglucanase precursor [Globodera rostochiensis] | 10 | 2.44E-126 | 66.53 |
| [BLASTED] | AHW98760.1 | beta-1,4-endoglucanase precursor [Globodera rostochiensis] | 10 | 3.64E-150 | 64.88 |
| [BLASTED] | AHW98758.1 | secreted SPRY domain-containing protein 4, partial [Globodera rostochiensis] | 2 | 8.66E-20 | 53.66 |
| [BLASTED] | ACO35734.1 | RBP-5 protein [Globodera pallida] | 2 | 5.33E-14 | 47.88 |
| [BLASTED] | ACO35733.1 | RBP-4 protein [Globodera pallida] | 2 | 6.22E-26 | 58.5 |
| [NO-BLAST] | ACO35732.1 | RBP-3 protein [Globodera pallida] |  |  |  |
| [BLASTED] | ACO35731.1 | RBP-2 protein [Globodera pallida] | 2 | 8.96E-19 | 49.24 |
| [NO-BLAST] | AHA80136.1 | CLAVATA3/ESR-related protein [Globodera tabacum solanacearum] |  |  |  |
| [NO-BLAST] | AHA80119.1 | CLAVATA3/ESR-related protein [Globodera tabacum tabacum] |  |  |  |
| [NO-BLAST] | AHB30320.1 | CLAVATA3/ESR-related protein [Globodera tabacum ssp. 'azteca'] |  |  |  |
| [NO-BLAST] | AHB30312.1 | CLAVATA3/ESR-related protein [Globodera tabacum solanacearum] |  |  |  |
| [NO-BLAST] | AHB30307.1 | CLAVATA3/ESR-related protein [Globodera tabacum tabacum] |  |  |  |
| [BLASTED] | pdb\|5OEV\|D | Chain D, Glutathione synthetase-like effector 22 (Gpa-GSS22-apo) | 10 | 1.60E-107 | 54.61 |
| [BLASTED] | pdb\|5OET\|A | Chain A, Glutathione synthetase-like effector 30 (Gpa-GSS30-apo) | 10 | 7.36E-73 | 50 |
| [BLASTED] | ACH56227.1 | cathepsin S-like cysteine proteinase | 10 | 0 | 56.58 |
| [BLASTED] | AIC75882.1 | serine carboxypeptidases | 5 | 0 | 73.97 |
| [BLASTED] | ADK46902.1 | Cathepsin-B | 10 | 0 | 66.03 |

**Table C. Presence or absence of signal peptide and the localization likelihood of *R. similis* cellulase genes**

| Gene_name | Localization | SignalP (yes or no) | CAZyme |
| --- | --- | --- | --- |
| Cellulase _contig_1188 | Extracellular | yes - NoTM | GH5_2 |
| Cellulase _contig_3276 | Cytoplasm | No | GH5_2 |
| Cellulase_ contig_2108 | Extracellular | yes - NoTM | GH5_2 |
| Cellulase _ contig_6969 | Extracellular | yes - NoTM | GH5_2 |
| Cellulase _ contig_3275 | Extracellular | yes - NoTM | GH5_2 |
| Cellulase _contig_1188 | Extracellular | yes - NoTM | GH5_2 |
| Cellulase_contig_482 | Extracellular | yes - NoTM | GH5_2 |
| Cellulase_contig_2617 | Extracellular | yes - NoTM | GH5_2 |
| Cellulase _contig_2204 | Extracellular | yes - NoTM | GH5_2 |
| Cellulase _ contig_1300 | Extracellular | yes - NoTM | GH5_2 |

**Table D. Localization patterns of *R. similis* pectate lyases (PL3)**

| Entry ID | Localization | Type | SignalP |
| --- | --- | --- | --- |
| PL3_contig_402 | Extracellular | Soluble | Yes-noTM |
| PL3_contig_1659 | Extracellular | Soluble | Yes-noTM |
| PL3_contig_403 | Cell membrane | Membrane | no |
| PL3_contig_1047 | Cell membrane | Membrane | Yes-noTM |

**Reference**

1. Li X, Yang D, Niu J, Zhao J, Jian H. De novo analysis of the transcriptome of Meloidogyne enterolobii to uncover potential target genes for biological control. International journal of molecular sciences. 2016;17: 1442.
