# Supplementary figures and images for: The genome of the migratory nematode, Radopholus similis, reveals signatures of close association to the sedentary cyst nematodes
Source: PLoS One. 2019 Oct 25;14(10):e0224391. doi: 10.1371/journal.pone.0224391 (PMC6814228; doi:10.1371/journal.pone.0224391)

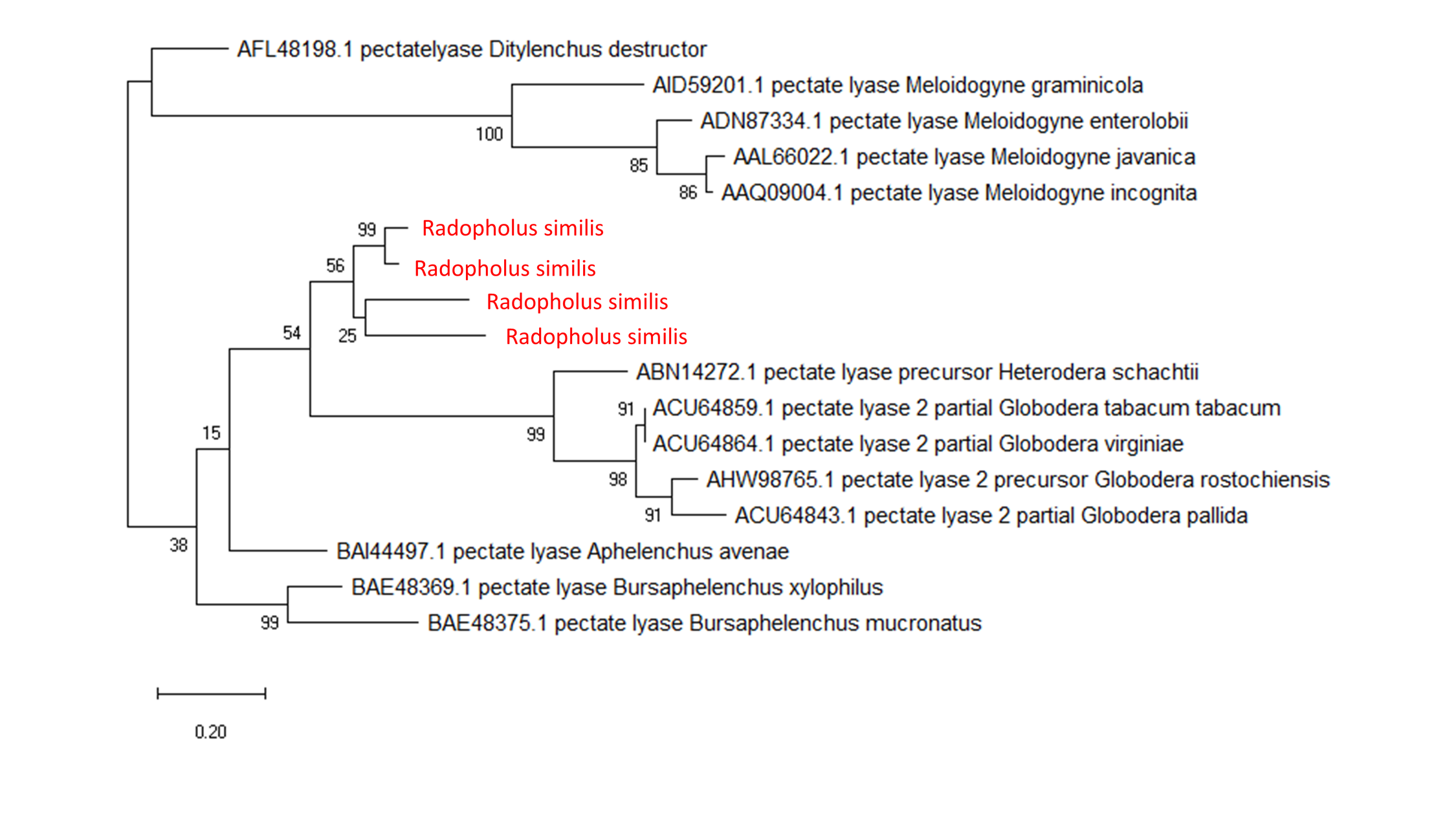

Supplement: S1 Fig — R. similis proteins are indicated in red. Genbank accession numbers are shown adjacent to each nematode. The numbers on the branches indicate bootstrap support value. 1000 bootstrap replications were performed. Scale indicates number of substitutions per site. (TIF) [file pone.0224391.s002.tif]

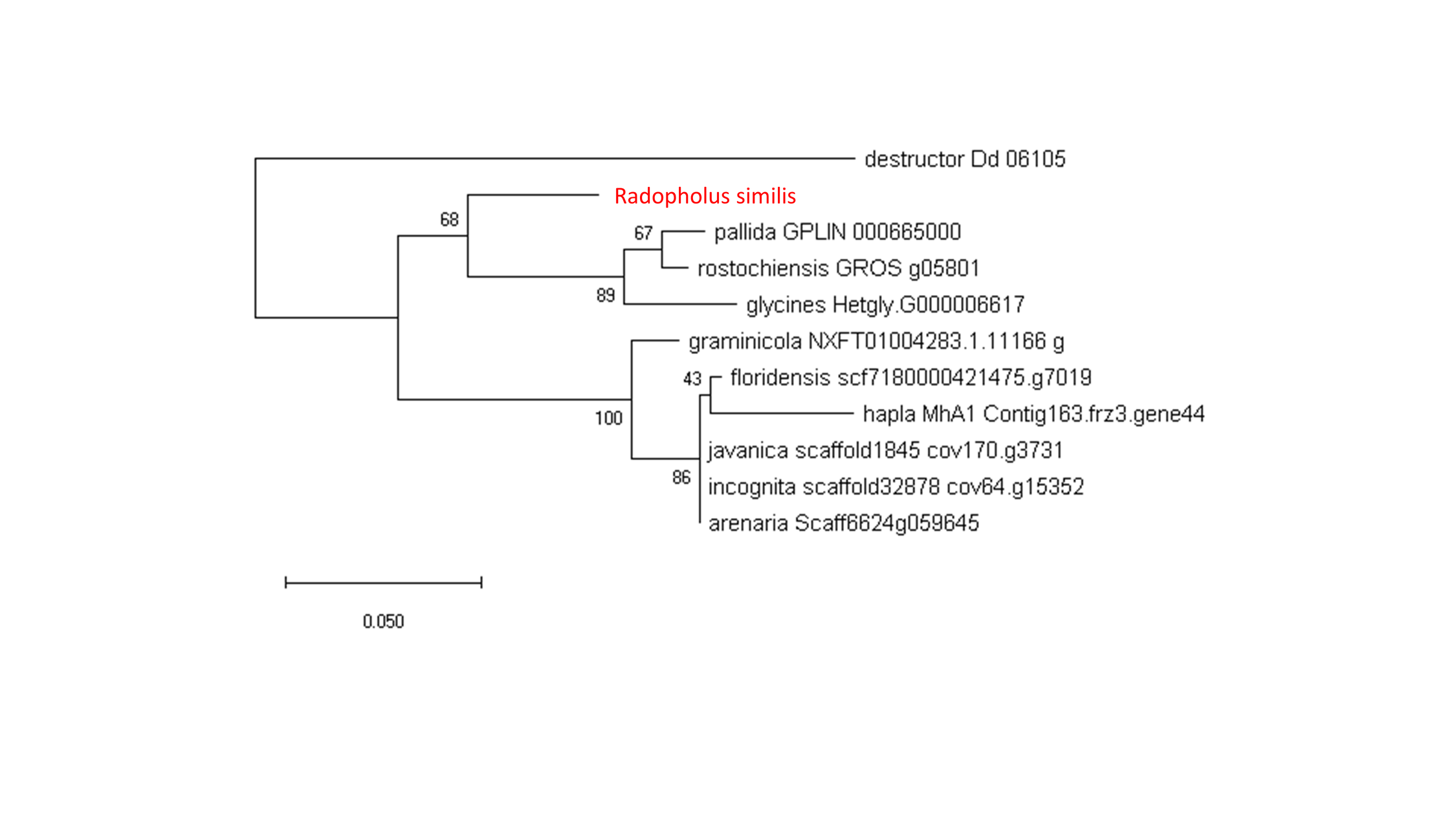

Supplement: S2 Fig — R. similis gpd gene is indicated in red. The numbers on the branches indicate bootstrap support value. 1000 bootstrap replications were performed. Scale indicates number of substitutions per site. (TIF) [file pone.0224391.s003.tif]

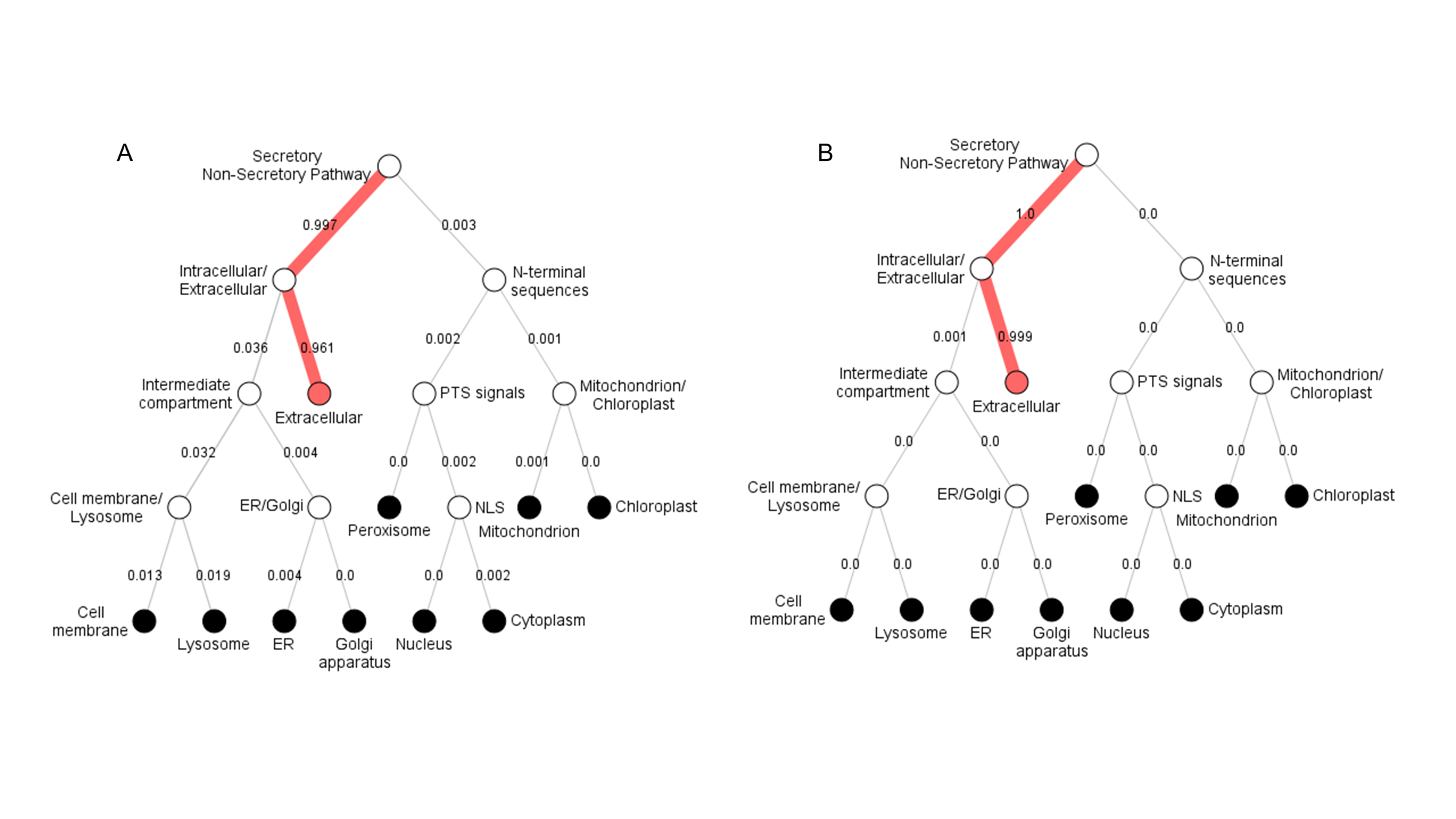

Supplement: S3 Fig — (A) R. similis predicted cellulase protein sequence and (B) H. glycines cellulase protein sequence (acc: AAC15707.1). Numbers on branches indicate localization likelihood in different compartments. Ideal localization path is highlighted in red. (TIF) [file pone.0224391.s004.tif]
